# Effectiveness and costs of non-invasive foetal RHD genotyping in rhesus-D negative mothers: a French multicentric two-arm study of 850 women

**DOI:** 10.1186/s12884-018-2114-5

**Published:** 2018-12-14

**Authors:** Meryl Darlington, Bruno Carbonne, Agnès Mailloux, Yves Brossard, Annie Levy-Mozziconacci, Anne Cortey, Hassani Maoulida, Tabassome Simon, Alexandra Rousseau, Isabelle Durand-Zaleski, the GENIFERH1 Study Group, Bruno Carbonne, Lionel Carbillon, Olivier Chanelles, Aurélie Revaux, Marc Dommergues, Anne-Florence Naime-Alix, Danièle Vauthier Brouzes, Christine Rouillac-Le Sciellour, Patrick Rozenberg, Valérie Serazin, Véronique Houfflin-Debarge, Stéphane Bezieau, Olivier Pape, Norbert Winer, Anne Cortey, Yves Brossard, Nelly Da Silva, Agnès Mailloux, Vassilis Tsatsaris, Sophie Omnès, Laurent Gavard, Laurent Mandelbrot, Claude d’Ercole, Raoul Desbrière, Jean-Baptiste Haumonté, Nathalie Lesavre, Annie Levy-Mozziconacci, Constance Borie, Aude Recoules-Arché, Yves Colin, Pascal Auquier, Cécile Fortanier, Sandrine Loubière, Abdel Benamar, Elodie Drouet, Mehdi Khadefi, Josiane Ngomlong, Alexandra Rousseau, Tabassome Simon, Sétareh Zarrabian, Meryl Darlington, Isabelle Durand-Zaleski, Hassani Maoulida

**Affiliations:** 1 AP-HP Health Economics Clinical Research Platform (URCEco), Hotel Dieu, Place de Parvis, 75004 Paris, France; 2 AP-HP, Hôpital Saint Antoine, 184 Rue du Faubourg Saint-Antoine, 75012 Paris, France; 3 Unité fonctionnelle biologique du centre national de référence en hémobiologie périnatale (CNRHP), Hôpital Saint Antoine, (AP-HP), 184 rue du Faubourg Saint-Antoine, 75012 Paris, France; 4 Unité fonctionnelle clinique du centre national de référence en hémobiologie périnatale (CNRHP), Hôpital Trousseau, (AP-HP), 26 avenue du Dr Arnold-Netter, 75012 Paris, France; 5 AP-HM, Hôpital Nord, Chemin des Bourrely, 13915 Marseille cedex 20, France; 6 AP-HP, Unité de Recherche Clinique de l’Est Parisien (URC-Est), Hôpital Saint Antoine, 184 Rue du Faubourg Saint-Antoine, 75012 Paris, France

**Keywords:** Anti-D immunoglobulin, Non-invasive foetal RHD genotyping, Cost evaluation, Cost-effectiveness

## Abstract

**Background:**

The determination of foetal Rhesus D (RHD) status allows appropriate use of IgRh prophylaxis by restricting its use to cases of RHD feto-maternal incompatibilities. There is a degree of uncertainty about the cost-effectiveness of foetal RHD determination, yet screening programs are being introduced into clinical practice in many countries.

This paper evaluates the impact of non-invasive foetal Rhesus D (RHD) status determination on the costs of managing RHD-negative pregnant women and on the appropriate use of anti-D prophylaxis in a large sample of RHD-negative pregnant women using individual prospectively collected clinical and economic data.

**Methods:**

A prospective two-armed trial of RHD negative pregnant women was performed in 11 French Obstetric Departments. Non-invasive foetal RHD genotyping was performed before 26 weeks' gestation in the experimental arm whereas the control arm participants received usual care. The costs associated with patient management in relation to their RHD negative status (biological tests, anti-D prophylaxis and visits) were calculated from inclusion to the end of the postpartum period. The costs of hospital admissions during pregnancy and delivery were also determined.

**Results:**

A total of 949 patients were included by 11 centres between 2009 and 2012, and 850 completed follow-up, including medical and biological monitoring. Patients were separated into two groups: the genotyping group (*n*=515) and the control group (*n*=335). The cost of the genotyping was estimated at 140 euros per test. The total mean cost per patient was estimated at €3,259 (SD ± 1,120) and €3,004 (SD ± 1,004) in the genotyping and control groups respectively. The cost of delivery represented three quarters of the total cost in both groups. The performance of managing appropriately RHD negative anti-D prophylaxis was 88% in the genotyping group, versus 65% in the control group. Using the costs related to RHD status (biological tests, anti-D immunoglobulin injections and visits) the incremental cost-effectiveness ratio (ICER) was calculated to be €578 for each percentage gain in women receiving appropriate management.

**Conclusion:**

Early knowledge of the RHD status of the foetus using non-invasive foetal RHD genotyping significantly improved the management of RHD negative pregnancies with a small increase in cost.

**Trial registration:**

Clinical trials registry-NCT00832962–13 January 2009 - retrospectively registered.

**Electronic supplementary material:**

The online version of this article (10.1186/s12884-018-2114-5) contains supplementary material, which is available to authorized users.

## Background

Pregnant RHD negative women are at risk for alloimmunization if the foetal blood group is RHD positive. RHD alloimmunization can be prevented by injection of anti-D specific immunoglobulin (IgRh), a plasma derived product, during pregnancy and after delivery. In 2005 the French College of Obstetricians and Gynaecologists (CNGOF) recommended systematic and targeted anti-D prophylaxis in all RHD negative women having a RHD positive partner. This policy involves injection of IgRh in three situations: following obstetrical events that can lead to feto-maternal haemorrhage (FMH), targeted after delivery if the newborn red blood cells are phenotyped as RHD positive and routine prevention at 28 weeks gestation to protect against spontaneous occult FMH of the third trimester. This policy has resulted in an estimated rate of 0.1 to 0.7% RHD alloimmunizations nationwide [1].

Knowledge of the foetal RHD status allows appropriate use of IgRh prophylaxis by restricting its use to cases of RHD feto-maternal incompatibilities. Non-invasive foetal RHD genotyping via the amplification of cell-free foetal DNA in the plasma of pregnant women also avoids the use of amniocentesis for foetal blood group genotyping.

Targeted prophylaxis, made possible by the knowledge of foetal RHD status by non-invasive genotyping, should reduce the quantity of IgRh injections and limit unnecessary exposure to IgRh, which is a human blood product. The additional cost of the genotyping should be in part covered by a reduction in IgRh administration and reduce overall expenses when compared to the systematic preventative administration of anti-D immunoglobulin at 28 weeks gestation.

In 2016 a systematic review reported the diagnostic accuracy and clinical effectiveness of RHD genotyping and estimated a false negative rate of 0.34% (95%CI 0.15 to 0.76) and a false positive rate of 3.86% (95%CI 2.54 to 5.82) suggesting that this test could reduce unnecessary use of anti-D with only a small increase in risk of alloimmunization [2]. The same systematic review included seven cost-effectiveness studies of which one was a one-armed prospective study (*n* = 101) in France [3] and foetal genotyping was not found to be an effective cost-reduction strategy.

However, foetal RHD screening programs are being introduced into clinical practice in countries like the UK, the Netherlands, Australia, so that only RHD negative mothers of RHD positive foetuses receive routine antenatal anti-D prophylaxis and the ethical case would seem to support the widespread use of foetal genotyping [4].

The aim of the present study was to evaluate the impact of non-invasive foetal *RHD* genotyping in a large sample of RHD negative pregnant women followed in University Hospital maternities in France in terms of cost, diagnostic accuracy and management of anti D prophylaxis practices.

## Methods

### Design and setting

This non-randomized open label multicentre prospective two-arm study was conducted between 2009 and 2012 in 11 maternities in France. The intervention (“genotyping”) group was recruited prospectively in 6 hospitals and the control group recruited in 5 hospitals both prospectively and retrospectively. The study period extended from the first prenatal visit to the end of the postpartum period.

### Patients

Patients in the foetal RHD genotyping arm were recruited if they fulfilled the following inclusion criteria: age above 18 years, pregnancy between 8 and 26 weeks, negative results to the Indirect Antiglobulin Test (IAT) at inclusion and RHD negative phenotype that had been determined using standard obstetric serological testing protocols. Women who had previously undergone an invasive foetal RHD genotyping, either by Chorionic villous sampling or by amniocentesis, were excluded.

Patients in the control arm (no foetal RHD genotyping) were recruited in two ways. Either prospectively as described for the genotyping arm above or selected by the National Reference Centre of Perinatal Hemobiology (CNRHP); RHD negative women were identified at the end of their pregnancy by the CNRHP who received the blood samples from the Centres in the control arm. The women concerned then signed a specific retrospective informed consent form. In the prospective genotyping arm, a RHD negative partner was an exclusion criterion. In the control arm, the partner’s RHD phenotype was not always reported in the file of the patient.

The study (NCT 00832962) was approved by a National Ethical Committee and all participants signed an informed consent form.

In the genotyping arm, maternal blood samples (2 × 4.5 mL EDTA) were collected from RHD negative pregnant women referred to maternity care centres from 12 weeks amenorrhea to ensure sufficient quantity of free foetal DNA. All test results were transmitted to the referring physicians/midwives to guide clinical and laboratory management of pregnancies.

Prevention with IgRh (Rhophylac® 300mcg) was planned at 28 weeks of gestation +/− 1 week, within 72 h post-delivery (postpartum) and as required in case of an adverse event at risk of FMH, such as abdominal trauma or vaginal bleeding, in accordance with the French guidelines for clinical practice. With the exception of the non-invasive genotyping in the experimental arm, the prenatal visits and biological tests for the all women participating were as standard practice and no additional visits were planned due to participation in this study.

### Tests

In 1997 it was demonstrated that free foetal DNA is present in maternal plasma and could be used for non-invasive prenatal diagnosis [5]. This paved the way for foetal RHD genotyping after the extraction and concentration of DNA from the plasma of RHD negative pregnant women. The methods involving real-time PCR, primers and probes targeted toward exons 7 and 10 of the RHD gene have been described elsewhere [6].

For our study, a first generation reagent kit - the Free DNA Fetal Kit® RHD – received CE marking in 2007 and was manufactured by the Institute of Biotechnology Jacques BOY in cooperation with the French National Institute of Health and Medical Research (INSERM) and the National Institute of Blood Transfusion (INTS) [7].

The sensitivity of the test is known to be high but this level of accuracy can be jeopardized by an insufficient quantity of foetal DNA, for example if the blood sample was taken before 10 weeks of gestation. When the foetus was found to be RHD negative at a first test, this result was systematically controlled by a second genotyping test on a new blood sample taken at least one week later and at least 15 weeks of gestation to prevent false negatives. A patient with first foetal test results of RHD negative would continue to be treated according to the guidelines as if RHD positive until the second test confirmed the negativity. An indeterminate first result would also lead to a new blood sample and a second test being carried out. In case of a twin pregnancy, the foetal genotyping result would be given without the possibility of identifying which of the two foetuses are RHD positive since the impact on immunoprophylaxis during the pregnancy and at delivery would be the same.

At the end of the pregnancy all of the mothers were checked for indirect Antiglobulin test (IAT) with microtitration to identify the presence of passive or immune anti-D if positive and a Kleihauer-Betke test to adjust the dose of IgRH required. The RHD status and the direct Antiglobulin test were performed on the blood of each newborn. These tests were carried out in accordance with the French CGNOF guidelines [1].

### Costs and economic evaluation

The prospective economic evaluation was conducted from the healthcare perspective to determine the cost per pregnancy at risk managed using foetal *RHD* genotyping compared to usual care. Healthcare resources included: hospital admissions during pregnancy, prenatal visits, IgRh injections, genotyping and other biological tests. The unit cost of genotyping was obtained with a bottom-up micro-costing approach that identified all relevant cost components of the process and valued each using the duration of the procedure, staff and supplies as variables. Costs of all other biological tests and clinic visits were based on current tariffs; cost of hospital admissions used the national cost study (Etude nationale des coûts ENC). All unit costs are presented in the supplementary material (Additional file 1).

Cost computations were based on healthcare resources actually used by participants during the trial, including office visits and tests up to and including the delivery. All IgRh utilisation was recorded. The initial timing for prevention was planned at 28 weeks of gestation +/− 1 week but in reality sometimes took place outside that window. Thus the analysis considered that any injection of IgRh between 26 and 32 weeks gestation that was not following an obstetrical event was prophylaxis. Non-healthcare resources were excluded. All costs were calculated at 2014 prices in € (1€ = 1.2US$) and not discounted due to the short time frame. The incremental cost effectiveness ratio (ICER) was calculated as incremental costs per additional woman appropriately treated. Appropriate treatment was defined by the sum of RHD negative women who were at risk and received prophylaxis and those who were not at risk and did not receive prophylaxis. Performance was defined as the percentage of RHD negative women receiving appropriate management. Our methodology was in accordance with the Consolidated Health Economic Evaluation Reporting Standards (CHEERS) statement.

Economic analyses compared the genotyping and control groups. Continuous variables were described using mean ± SD and qualitative variables were described using percentages. Differences in costs were tested using standard parametric (Students) or nonparametric (Mann-Whitney’s test or Bootstrap resampling) as appropriate and were described using mean ± SD. The *p* values are two-sided with a significance level of < 0.05. A joint comparison of costs and effects was performed by bootstrapping with 1000 resamples and the results of the bootstrap replications presented on a cost-effectiveness plane and acceptability curves. SAS (Version 9.3, SAS Institute, Cary, NC) was used for all analyses.

## Results

**Fig. 1:**
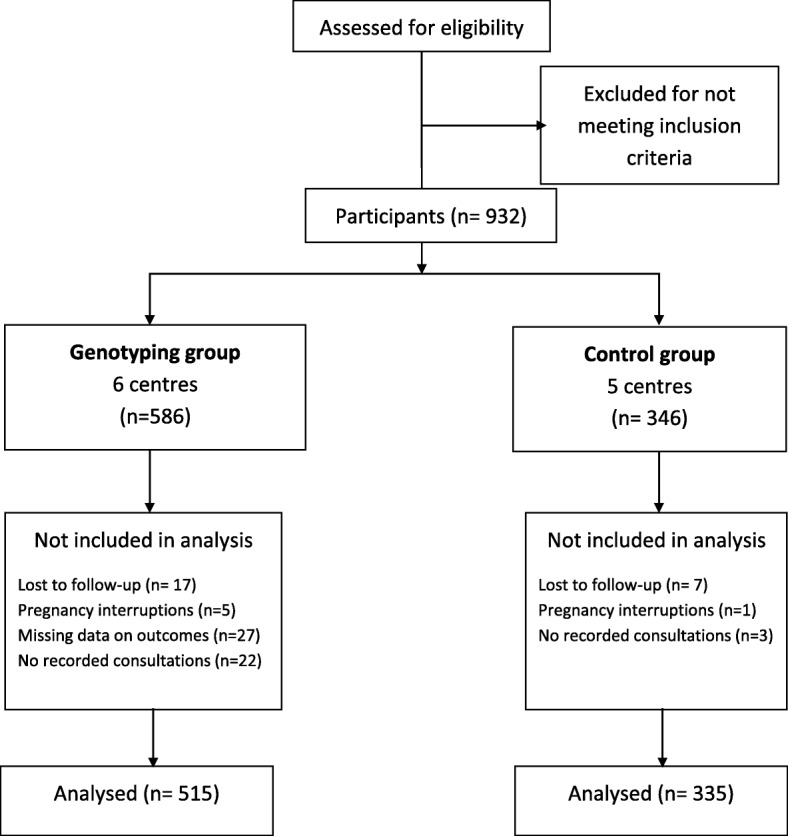
Study flow chart

A total of 949 patients were included in 11 centres between 2009 and 2012, and 850 completed follow up, 515 in the genotyping group and 335 in the control group (Fig. 1). For the 850 included in the analysis, the average monitoring time up to the end of pregnancy was 24.8 (SD ± 7.7) weeks in the genotyping group and 25.4 (SD ± 8.1) weeks in the control group (Table 1).

**Table 1:** Population characteristics

Characteristics	Genotyping *n* = 515	Controls *n* = 335	Total population *n* = 850
Age (mean ± sd in years)	30 ± 5	31 ± 5	31 ± 5
Gestational age at inclusion (mean ± sd in weeks)	19 ± 4	28 ± 9	23 ± 8
Type of pregnancy at inclusion			
Singleton	499 (97%)	325 (97%)	824 (97%)
Twins	16 (3%)	10 (3%)	26 (3%)
Amniocentesis genotyping result	17 (3%)	2 (1%)	19 (2%)
Outcome of pregnancy			
Delivery	512 (99%)	331 (98%)	843 (99%)
in utero foetal death	3 (< 1%)	1 (< 1%)	4 (< 1%)
Missing information	0 (0%)	3 (1%)	3 (< 1%)
Newborn phenotype			
RHD positive	368 (71%)	212 (63%)	580 (68%)
RHD negative	136 (26%)	100 (30%)	236 (28%)
Undetermined or missing data	11 (2%)	23 (7%)	34 (4%)
Father phenotype			
RHD positive	376 (73%)	126 (38%)	502 (59%)
RHD negative	19 (4%)	19 (6%)	38 (4%)
Undetermined or missing data	120 (23%)	190 (57%)	310 (36%)
Type of delivery N (%)			
Vaginal	395 (77%)	264 (79%)	659 (77%)
Caesarean section during labour	62 (12%)	37 (11%)	99 (12%)
Elective caesarean section	56 (11%)	34 (10%)	90 (11%)
Missing information	2 (< 1%)	0 (0%)	2 (< 1%)

At inclusion, none of the participants had undergone either an invasive or non-invasive genotyping procedure. During the study, of the 515 women included in the genotyping group, 368 (71%) were positive and therefore at risk of immunization, 136 (26%) were negative and should not receive IgRh prophylaxis and 11 (2%) were undetermined or incorrectly documented. In the control group, two women (1%) had RHD genotyping during amniocentesis. During the course of their pregnancy, 113 (22%) women in the genotyping group and 66 (20%) in the control group experienced an event resulting potentially in FMH (miscarriage, bleeding, amniocentesis, *cervical cerclage*). There were 91 (18%) women requiring a pregnancy-related hospital admission in the genotyping group versus 65 (19%) in the control group.

In the genotyping group a total of 336 (65%) women received IgRh prophylaxis between weeks 26 and 32 of gestation, 113 (22%) received an injection at a different time, either due to an event or mistiming of the prophylaxis period, and 354 (69%) received a post-partum injection. In the control group 261 (78%) women received IgRh prophylaxis between weeks 26 and 32 gestation, 74 (22%) received an injection at a different time, either due to an event or mistiming of the prophylaxis period and 204 (61%) a post-partum injection (Table 2). The partner’s RHD phenotype was not documented for 120 (23%) patients in the genotyping arm and 190 (57%) in the control arm. We observed an obstetric event rate in our study of 22% which is comparable with the Benachi [3] study that reported an obstetric rate of 23%.

**Table 2:** IgRh prophylaxis summary

Newborn phenotype	RHD +		RHD -		ND		Total	
Genotyping arm								
Number of patients	368	(100%)	136	(100%)	11	(100%)	515	(100%)
At least one injection	366	(99%)	24	(18%)	8	(73%)	397	(77%)
prophylaxis	*320*	*(87%)*	*10*	*(7%)*	*6*	*(55%)*	*336*	*(65%)*
obstetrical event	*95*	*(26%)*	*16*	*(12%)*	*2*	*(18%)*	*113*	*(22%)*
postpartum	*346*	*(94%)*	*1*	*(1%)*	*8*	*(73%)*	*354*	*(69%)*
No injection	2	(1%)	112	(82%)	3	(27%)	118	(23%)
Control arm								
Number of patients	212	(100%)	100	(100%)	23	(100%)	335	(100%)
At least one injection	211	(99.5%)	85	(85%)	19	(83%)	315	(94%)
prophylaxis	*174*	*(82%)*	*73*	*(73%)*	*14*	*(61%)*	*261*	*(78%)*
obstetrical event	*50*	*(24%)*	*19*	*(19%)*	*5*	*(22%)*	*74*	*(22%)*
postpartum	*198*	*(93%)*	*2*	*(2%)*	*4*	*(17%)*	*204*	*(61%)*
No injection	1	(0.5%)	15	(15%)	4	(17%)	20	(6%)

RHD phenotype was determined after delivery for 504 (98%) and 312 (93%) newborns in the genotyping and control groups respectively. Out of these, 368 (73%) in the genotyping group and 212 (63%) in the control group were RHD positive.

In this study, foetal RHD genotyping early during pregnancy, using a second genotyping test to control a RHD negative result, ensured that 87% of RHD negative women with RHD positive babies receive IgRh prophylaxis (compared to 82% in the control arm) and that 93% of RHD negative mothers with RHD negative babies avoid unnecessary IgRh injections compared to 27% in the control arm. For 10 of the patients in the genotyping arm, a third test was performed.

The sensitivity of the genotyping test, not including retesting of negative results, was 98.1% and the specificity was 88.2%. The risk of a false negative result was minimized by controlling the first genotyping results with a second test on a new blood sample and this strategy resulted in a sensitivity of 99.7% and a specificity of 92.6%. For the first round of tests, the inconclusive genotyping results accounted for 7.6% of the tests. The addition of the second test for RHD negative or inconclusive test results lowered this undetermined rate to 1.6% and these patients were treated as RHD positive.

For two mothers of babies with a RHD positive phenotype at birth who did not have records of receiving any IgRh injection during pregnancy, one had a false negative genotyping result, which was taken into consideration incorrectly post-partum rather than using the phenotyping result and one had missing data. Nine of ten mothers with babies with a phenotype of RHD negative at birth, who received an IgRh injection at 28 weeks, had false positive genotyping results.

Effectiveness, or performance, was the sum of the number of RHD negative women who were at risk and received prophylaxis and women who were not at risk and did not receive prophylaxis as a fraction of the total population. When only the first test was considered, effectiveness in the genotyping group was 85% versus 62% in the control group *(p* < 0.0001); using information from the 2 tests increased only marginally the performance in both groups, to 88% in the genotyping group versus 63% in the control group.

Healthcare resource use and costs are presented in Table 3. When all direct healthcare costs were included, the genotyping arm cost per patient was on average €255 more expensive than the control arm. The cost of genotyping of €140 includes the cost of the commercial kit, all other materials and human resource costs. When the cost analysis was limited to resources pertaining to RHD negative status, the cost difference was reduced to €139.

**Table 3:** Average cost per woman in € included in the study from the time of inclusion to the end of the follow-up period at delivery

Cost centre	Genotyping	Controls	*p* value
	n = 515	n = 335	
Costs relating to RHD - status			
Biological tests - Genotyping, IAT, Kleihauer	268 ± 74	105 ± 49	< 0,001
Anti-D immunoglobulin injections	136 ± 86	136 ± 57	0.94
Consultations	187 ± 79	211 ± 76	< 0,001
Total costs related to RHD status	591 ± 113	452 ± 124	< 0,001
Hospital stay costs			
Hospital admissions during pregnancy	262 ± 910	280 ± 844	0.36
Delivery	2406 ± 469	2272 ± 477	< 0,001
Total costs relating to hospital stays	2668 ± 1101	2552 ± 984	0.95

When only one test was carried out even in the case of RHD negative results, the average cost per patient of genotyping decreased by a further €49, leading to a cost difference between the arms of €90. Replacing the commercial kit with the cost of reagents available to the hospital purchasing department, the cost average cost per patient of genotyping would decrease by a further 51 euros leading to a cost difference between the two arms of €39. We used the results based on retesting to confirm an RHD negative foetus in the cost-effectiveness analysis to calculate the incremental cost effectiveness ratio and estimated €578 for a 1 % increase in the number of women receiving appropriate management as show in Table 4. This means that for one extra person to receive the appropriate IgRh prophylaxis, a total of 4 women would have to undergo foetal genotyping.

**Table 4:** Incremental cost-effectiveness results

Intervention n	Average cost (€)	Performance ^a^ (%)	∆ Cost (€)	∆ Performance^a^ (%)	ICER ∆ Cost / ∆ Performance^a^
Based on costs related to RHD status (tests, anti-D injections and visits)					
Usual care	452	64%			
RHD genotyping	591	88%	139	24%	578

^a^ *Performance is defined as the percentage of RHD negative women receiving appropriate management*

**Fig. 2:**
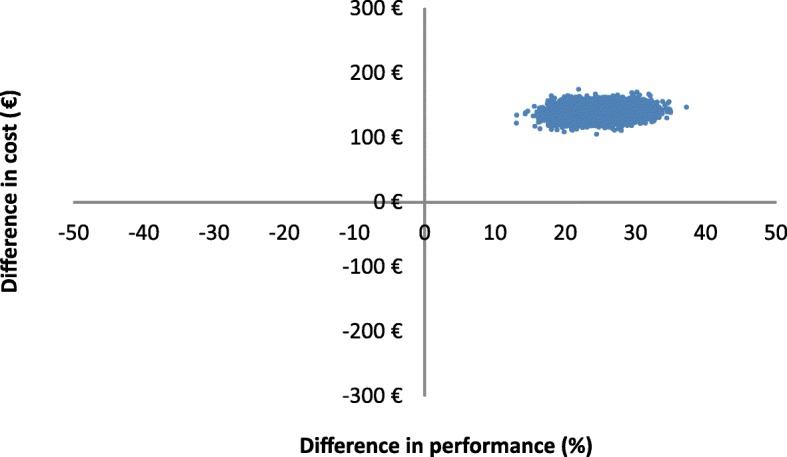
Cost-effectiveness plane. Scatter plot on the cost-effectiveness plane showing the difference in costs (pertaining to RHD negative status) and performance from GENIFERH1 data using 1000 bootstrap replicates. The genotyping arm cost of resources per patient was on average €139 more expensive than the control arm. The genotyping arm was more performant with an increase in effectiveness of 24%

**Fig. 3:**
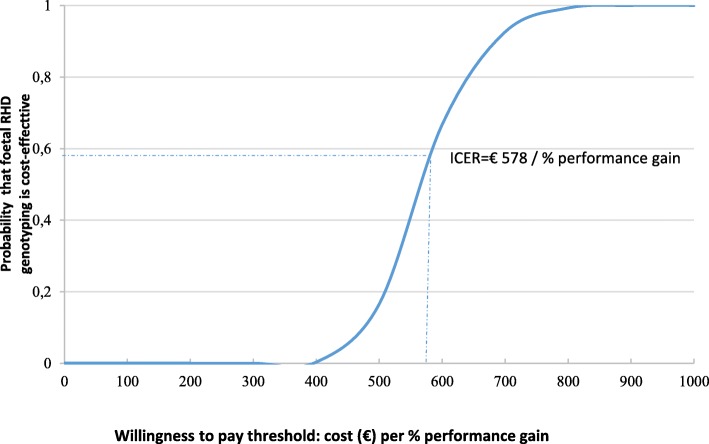
Cost-effectiveness acceptability curve showing the probability that foetal RHD genotyping is cost-effective compared to usual care based on the uncertainty in cost and effect differences shown in Fig. 2

The results of the bootstrap analysis are shown as a scatter plot of 1000 ICERs presented on the cost-effectiveness plane (Fig. 2). The horizontal axis shows the difference in performance between the genotyping group and the control group. The vertical axis represents the cost difference between the two strategies. The scatter plot is contained in the north-east quadrant. In other words, foetal genotyping is a cost-increasing/quality-increasing innovation. There is no fixed willingness to pay threshold in France but at a theoretic threshold of €585 / percentage gain in performance, there is a 60% chance that foetal RHD genotyping is cost effective. We have also represented the results of the bootstrap analysis in a cost effectiveness acceptability curve in Fig. 3.

The economic results in Table 3 and in Figs. 2 and 3 are based upon the costs related to RHD status (biological tests, anti-D immunoglobulin injections and visits). The supplementary material (Additional file 2) shows the results taking into consideration all direct health costs measured during the trial including all hospital stays.

## Discussion

Non-invasive foetal RHD genotyping has been implemented as standard practice in some European countries. Whether the use of this assay should be extended to all RHD negative pregnant women is still debated. In this controlled study of 850 patients, we found that the realization of systematic non-invasive foetal RHD genotype in all RHD negative pregnant women with IAT negative test results (not immunized), increased from 63 to 88% the rate of appropriate IgRh prophylaxis at 28 weeks of gestation.

The NICE guidance of 2016 states that the test is highly accurate after 11 weeks of gestation. It is possible that by carrying out the test as early as 10 weeks gestation, the sensitivity would have been less given the low concentrations of cell-free foetal DNA in early pregnancy and that this could have led to some Rh + babies going undetected. However, the protocol specified that a second test should be carried out to confirm RH- status of the foetus at 15 weeks so the overall sensitivity of the strategy would not have been affected.

The primary analysis in our study, with a strategy of controlling the RHD negative results, resulted in a sensitivity of 99.7% and a specificity of 92.6%. The false negative rate of 0.3% is very similar to the results of the NICE systematic review. The percentages of incorrect test results were very similar between the NICE systematic review (2%) and our study (4.6% for one genotyping test, 2% for the strategy of controlling RHD negative results). The effectiveness of our study of 88% is comparable to the range reported in the NICE report of 86 to 96%. The percentage of undetermined genotyping results in the NICE systematic review of 4 to 7% was similar to our study with 8.2% undetermined after one genotyping only and 1.6% with the strategy of confirming RDH negative results. Our results suggest that use of genotyping to determine IgRh use would substantially reduce the number of women receiving IgRh prophylaxis unnecessarily from 77 to 7%.

We observed that only 87% of RHD negative women with RHD positive babies received IgRh prophylaxis in the experimental arm and 82% in the control arm. Despite the 2005 guidelines, many obstetricians were still reticent about prenatal prophylaxis in 2009 as documented in a report by the CNGOF and they observed a large diversity in prophylaxis at the national level. In addition, the risk of exposure to an infectious agent in the immunoglobulin derived from blood was cited as a negative factor. These issues may have affected the adherence to guidelines and may explain the 82–87% rate of prenatal prophylaxis in our study [8].

One limit of our study was the uncertainty about the fathers RHD status in the control arm, which may have led to more RHD negative fathers in the control arm and would account for the percentage of RHD negative newborns in the control arm being higher than in the genotyping arm, 32% versus 27%. This would have led to a higher percentage of IgRh injections in the genotyping arm than would be expected since the arms were not equivalent in terms of paternal RHD status. In the primary analysis we have not corrected for these differences and the average number of IgRh injections is the same in both arms at 1.6 injections on average per woman. In addition 22% (*n* = 74) of women in the control arm did not have a record of receiving the systematic prophylaxis injection at 28 weeks, which in turn lowered the average cost of injections in the control arm. These deviations from the French national guidelines and the protocol had the effect of raising the relative average costs in the genotyping arm when compared to the control arm. Thus the additional cost of the test was not offset by the reduction of IgRh injections and the cost of follow-up in the genotyping arm was on average €255 more expensive than in the control arm if all costs including delivery and hospitalizations are considered. When the cost analysis was limited to resources pertaining to RHD negative status, the cost difference was reduced to €139. Other authors reported similar results by using a €150 unit cost per assay [3]**.** It is possible to use reagents that are mixed locally in the biology laboratories at a much lower cost than the commercial kit. Using the current tariffs available to public hospitals in Paris the cost of genotyping using the reagent mix would be €88 which is considerably less than the €140 cost of the commercial kit and would thus cut costs and allow greater flexibility and rapidity for including changes in the scope or quality of the test. However, if genotyping is to be widely implemented, a home-based mix may not be a viable solution due to potential reproductability issues.

Ten mothers with RHD negative babies correctly detected by genotyping did receive IgRh prophylaxis at 28 weeks gestation but this was because they had not received the genotyping results in time. The inclusion of patients could be carried-out up to 26 weeks of amenorrhea and the results of genotyping may not have been available at the time of routine prophylaxis in all cases. The possibility of carrying out the genotyping earlier in the pregnancy with improved techniques for DNA yield and PCR methods should improve the efficiency and prevent unnecessary administration of IgRh [9]. There is also the possibility that given that the study was run in real-life situations, the physician or midwife in charge of the follow-up may not yet have complete confidence in the results of this relatively new test.

More than 40,000 Rhesus negative women per year in France have RHD negative babies and thus are not at risk of alloimmunization [10]. Ensuring that these women do not receive a blood product unnecessarily would also be consistent with ethical priorities such as reducing wasteful use of such products [4].

## Conclusions

Early knowledge of the RHD status of the foetus using non-invasive foetal RHD genotyping significantly improved the management of RHD negative pregnancies. Using the test in a systematic way for all RHD negative pregnant women to ensure appropriate IgRh prophylaxis was shown to be cost increasing at €578 per percentage gain in women benefitting from correct prophylaxis. These results reflect what actually took place in the GENIFERH1 study based on a large sample (*n* = 850) with prospectively collected economic data. Whilst costs may be reduced through different strategies, our results confirm other studies’ findings that this is not an effective cost-reduction strategy. However, in all cases, there are clear clinical and ethical benefits in implementing this test in terms of better management of pregnant RHD negative patients and appropriate IgRh prophylaxis.

## Additional files

Additional file 1: Unit costs used in the cost analysis. (PDF 273 kb)

Additional file 2: Economic results using all direct medical costs collected during the trial. This supplementary material shows the economic analysis of the ICER, the cost-effectiveness plane and the acceptability curve that have been recalculated using all direct health costs (those pertaining to RHD status: biological tests, injections and visits, plus all hospital stays during the follow-up period). (PDF 358 kb)

## Abbreviations

CE: European Conformity
CHEERS: Consolidated Health Economic Evaluation Reporting Standards
CNGOF: French college of gynaecology and obstetrics
CNRHP: Centre National de Référence en Hémobiologie Périnatale
DNA: Deoxyribonucleic acid
EDTA: Ethylene diamine tetraacetic acid
ENC: French National Cost Study
FMH: Feto-maternal haemorrhages
IAT: Indirect Antiglobulin Test
ICER: Incremental cost-effectiveness ratio
IgRh: Anti-D specific immunoglobulin
INSERM: French National Institute of Health and Medical Research
INTS: National Institute of Blood Transfusion
RHD: Rhesus D
SD: Standard Deviation
