# Supplementary material for: Effectiveness and costs of non-invasive foetal RHD genotyping in rhesus-D negative mothers: a French multicentric two-arm study of 850 women
Source: BMC Pregnancy Childbirth. 2018 Dec 14;18:496. doi: 10.1186/s12884-018-2114-5 (PMC6295078; doi:10.1186/s12884-018-2114-5)
Supplement: Supplementary file 1 — Unit costs used in the cost analysis. (PDF 273 kb) [file 12884_2018_2114_MOESM1_ESM.pdf]

Table Unit Costs and Data Sources

Description		Unit cost €	Source of unit cost
**Biological tests** - Quantity and date of tests recorded in the CRF[1]			
	Genotyping	140,09	Micro-costing observations (all production costs included)
	Indirect Coombs - qualitative	10,08	Tarif NABM[2] http://www.codage.ext.cnamts.fr/codif/nabm/
	Indirect Coombs - quantitative	55,35	
	Kleihauer-Betke	18,90	
	Phenotyping test	20,25	
**Anti-D immunoglobulin injections** - Dose recorded in the CRF[1]			
	Rhophylac 200 µl	60,89	Pharmacy sales prices
	Rhophylac 300 µl	84,62	
**Consultations** - Practioner and type of visit recorded in the CRF[1]			
	Nurse (blood sample)	4,73	Tariff NGAP[3] http://www.ameli.fr/
	Nurse (injection)	6,30	
	Doctor	28,00	
	Midwife	23,00	
**Hospital costs** - Length of stays, parity, type & clinical detail recorded in event database and CRF[1]			
	During pregnancy	Variable DRG tariffs	DRG[4] tariff http://www.atih.sante.fr/
	Delivery		

1 CRF Case Report Form

2 NABM National Biology Database (Nomenclature des Actes de Biologie Médicale)

3 NGAP Profesionnal Medical Acts Database (Nomenclature générale des actes professionnels)

4 DRG Diagnosis Related Groups
