# Supplementary material for: Effectiveness and costs of non-invasive foetal RHD genotyping in rhesus-D negative mothers: a French multicentric two-arm study of 850 women
Source: BMC Pregnancy Childbirth. 2018 Dec 14;18:496. doi: 10.1186/s12884-018-2114-5 (PMC6295078; doi:10.1186/s12884-018-2114-5)

Supplementary Material - Economic results using all direct medical costs collected during the trial

(Biological tests, anti-D immunoglobulin injections, visits and hospital admissions)

Incremental cost-effectiveness results

**Intervention**	**Average cost (€)**	**Performance * (%)**	**Δ Cost (€)**	**Δ Performance * (%)**	**ICER Δ Cost / Δ Performance ***
Based on costs related to both RHD status (tests, anti-D injections and visits) and hospital stays (for delivery and other admissions during pregnancy)					
Usual care	3 004	64%			
RHD genotyping	3 259	88%	255	24%	1 059

** Performance is defined as the percentage ofRHD genative women receiving appropriate management*

Cost-effectiveness plane

Scatter plot on the cost-effectiveness plane showing the difference in costs (including all hospital admissions) and performance from GENIFERH1 data using 1,000 bootstrap replicates. The genotyping arm cost of resources per patient was on average €255 more expensive than the control arm. The genotyping arm was more performant with an increase in effectiveness of 24%.

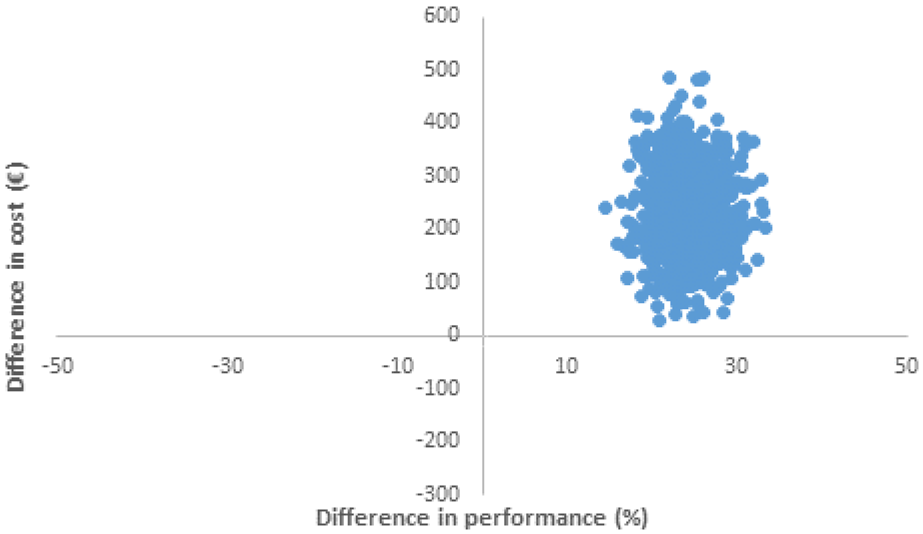

Cost-effectiveness acceptability curve

Cost-effectiveness acceptability curve showing the probability that foetal RHD genotyping is cost- effective compared to usual care using all direct costs recorded including <u>hospital stays</u>.

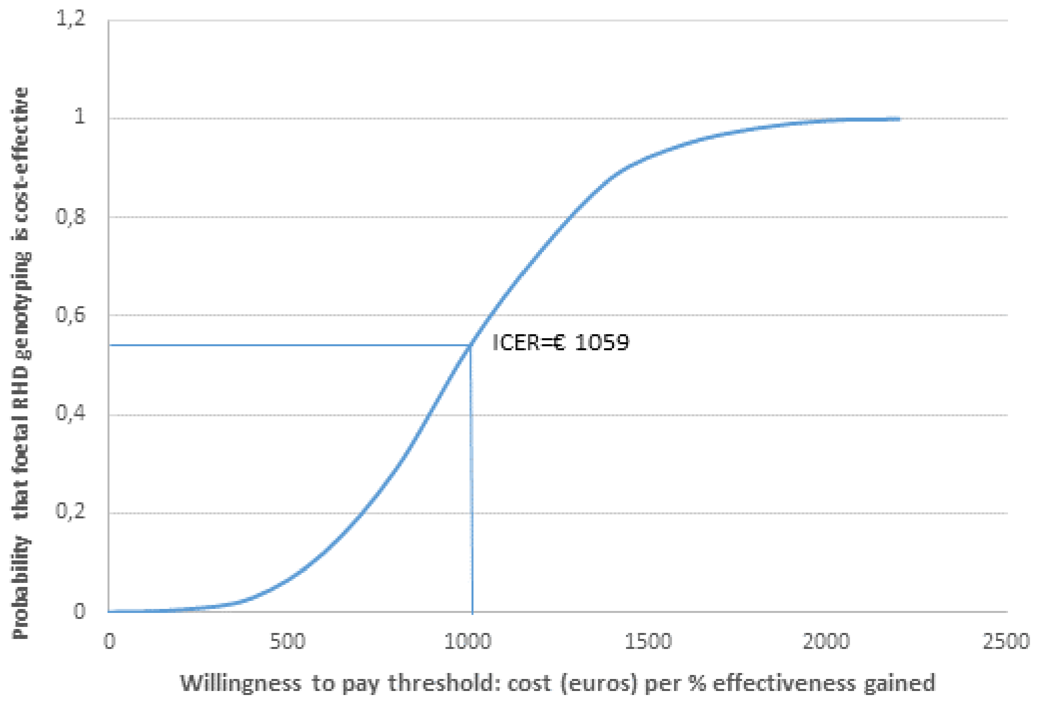

Supplement: Supplementary file 2 — Economic results using all direct medical costs collected during the trial. This supplementary material shows the economic analysis of the ICER, the cost-effectiveness plane and the acceptability curve that have been recalculated using all direct health costs (those pertaining to RHD status: biological tests, injections and visits, plus all hospital stays during the follow-up period). (PDF 358 kb) [file 12884_2018_2114_MOESM2_ESM.pdf]
